# Pyrrolidine-Derived Phenanthroline Diamides: An Influence of Fluorine Atoms on the Coordination of Lu(III) and Some Other f-Elements and Their Solvent Extraction

**DOI:** 10.3390/ijms24065569

**Published:** 2023-03-14

**Authors:** Nane A. Avagyan, Pavel S. Lemport, Mariia V. Evsiunina, Petr I. Matveev, Svetlana A. Aksenova, Yulia V. Nelyubina, Alexandr V. Yatsenko, Viktor A. Tafeenko, Vladimir G. Petrov, Yuri A. Ustynyuk, Xihe Bi, Valentine G. Nenajdenko

**Affiliations:** 1 Department of Chemistry, Lomonosov Moscow State University, 119991 Moscow, Russia; 2 A.N. Nesmeyanov Institute of Organoelement Compounds, Russian Academy of Sciences, 119334 Moscow, Russia; 3 Moscow Institute of Physics and Technology, National Research University, Institutskiy per. 9, 141700 Dolgoprudny, Russia; 4 Department of Chemistry, Northeast Normal University, 5286 Renmin Street, Changchun 130024, China

**Keywords:** phenanthroline, ligand, complex, lanthanide, lutetium, coordination number, XRD, UV-vis titration, NMR

## Abstract

Three pyrrolidine-derived phenanthroline diamides were studied as ligands for lutetium trinitrate. The structural features of the complexes have been studied using various spectral methods and X-ray. The presence of halogen atoms in the structure of phenanthroline ligands has a significant impact on both the coordination number of lutetium and the number of solvate water molecules in the internal coordination sphere. The stability constants of complexes with La(NO_3_)_3_, Nd(NO_3_)_3_, Eu(NO_3_)_3_, and Lu(NO_3_)_3_ were measured to demonstrate higher efficiency of fluorinated ligands. NMR titration was performed for this ligand, and it was found that complexation with lutetium leads to an approximately 13 ppm shift of the corresponding signal in the ^19^F NMR spectrum. The possibility of formation of a polymeric oxo-complex of this ligand with lutetium nitrate was demonstrated. Experiments on the liquid–liquid extraction of Am(III) and Ln(III) nitrates were carried out to demonstrate advantageous features of chlorinated and fluorinated pyrrolidine diamides.

## 1. Introduction

Lutetium is the heaviest and the smallest element of all the lanthanides [1,2]. Natural lutetium exists in two isotopic forms—in the form of a stable isotope ^175^Lu and a radioactive isotope ^176^Lu, which is used for radioisotope dating [3]. ^177^Lu is one of the most attractive isotopes in clinical nuclear medicine for the diagnosis and therapy of cancer [4].

A regular decrease in the ionic radii is observed in the lanthanide row. This effect, called “lanthanide contraction”, has been well studied and documented [5,6,7,8,9]. A variety of ligands have been shown to form complexes with lanthanides and the importance of lanthanide contraction on the structure of the formed complexes has been demonstrated [10,11,12]. Recently, we observed the effect of lanthanide contraction on tetrabutyl 1,10-phenanthroline-2,9-diamide [13], which acts as an N,N,O,O-tetradentate ligand. We revealed that in the La, Nd, and Eu complexes, metal ions are located below the plane of the phenanthroline core and the coordination number of the metal in the complexes with the ligand is 10. In the lutetium complex, the Lu^3+^ ion is located almost in the plane of the nucleus. As a result, the coordination number in the complex with lutetium decreases to nine and one of the nitrates became a monodentate. We have obtained a complex of lutetium nitrate with similar ligand-containing chlorine atoms at positions four and seven of the phenanthroline core. In this case, the coordination number of lutetium was also equal to nine. However, one of the nitrate groups was replaced with a more compact water molecule. The complex has been built on the principle of a tight ion pair having one nitrate group in the external coordination sphere [14].

Pyrrolidine-derived diamides are more compact ligands in comparison with tetrabutyl ligands. As a result, valuable properties of these ligands during the extraction separation of *f*-elements have been demonstrated [14,15,16,17]. Both the selectivity and extraction ability of the ligands under study decrease with the expansion of alicycle in the amide function despite an increase in solubility. These unusual properties can be explained by the molecular dynamics of such compounds [18,19,20,21,22].

It was shown in [17] that the presence of chlorine atoms in positions four and seven of the phenanthroline core and the variation of the structure of amide substituents may strongly affect the extraction properties of the ligands toward separation of lanthanides. At the same time, the replacement of chlorine atoms with fluorine allows us to expect increased resistance of ligands toward radiolysis in comparison with the chlorine-containing ligands. Recently, we reported on the effective synthesis of fluorinated phenanthrolinediamides [23]. A detailed comparison of the coordination properties of pyrrolidine-derived phenanthroline diamides is of significant interest. This study is devoted to a comparison of three phenanthroline-derived ligands **L1**–**L3** toward formation of lutetium complexes.

## 2. Results and Discussion

### 2.1. Structure of Ligands and Complexes

**Figure 1:**
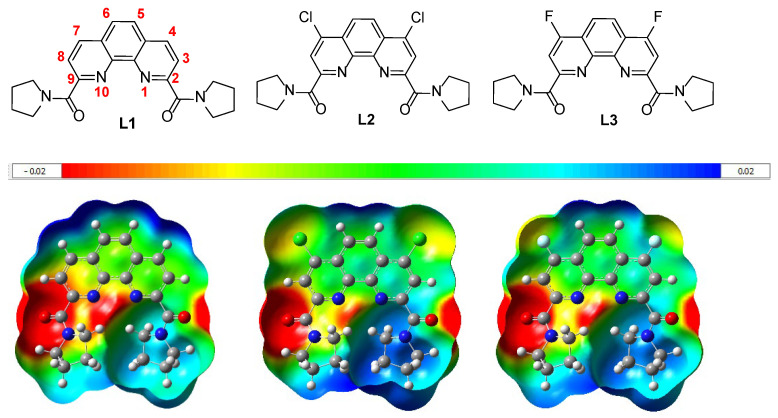

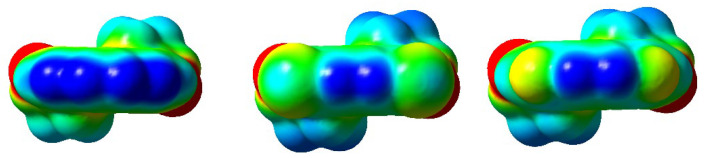
ESP maps of ligands **L1**–**L3** in two projections.

The ligands **L1**–**L3** have significant differences in their electronic properties (Figure 1). Calculated electrostatic potential maps (ESP maps) of ligands **L1**–**L3** permit the visualization of differences in electron distribution, which can be explained by the electron withdrawing influence of chlorine and fluorine.

Another possible way to view the electronic distribution in the ligands **L1**–**L3** is to use Merz-Kollman (ESP) charges [24]. For example, a difference in the corresponding charges on the nitrogen and oxygen atoms was observed for ligands **L1**–**L3** according to the calculated data (Table 1). The differences in the charges on nitrogen and oxygen atoms are due to the fact that the ligand structures are asymmetric. Ligands of this type exist in twisted conformations (Figure 1, a single potential scale from −0.02 to +0.02 conventional units). All quantum chemical calculations were performed by DFT with functional—B3LYP and basis—6-31G (d,p), using the Gaussian 16 program [25] method. The calculated ESP charges in carbonyl oxygen and phenanthroline nitrogen atoms in ligands **L1** and **L3** look very similar; small differences in the values of the corresponding charges are observed only for the ligand **L2** (Table 1).

It is quite remarkable that the negative charges on the nitrogen atoms of the phenanthroline core are the highest in the **L3** ligand, while the charges on the oxygen atoms in **L3** are somewhat lower than in **L1**, but higher than in **L2**. As is well known, fluorine is a strong σ-electron acceptor, but at the same time a good π-donor. The effect of direct polar conjugation manifests itself most clearly on nitrogen atoms in *para*-positions with respect to fluorine atoms, which is reflected in the stability constants of complexes with lanthanide cations (see below).

**Figure 2:**
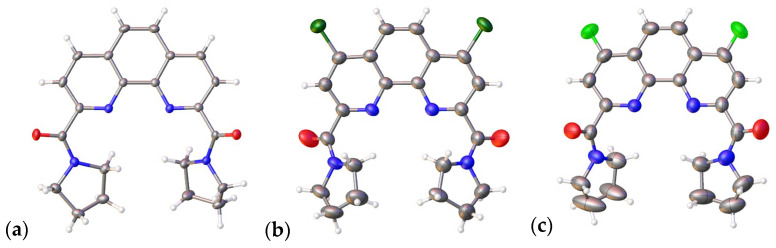

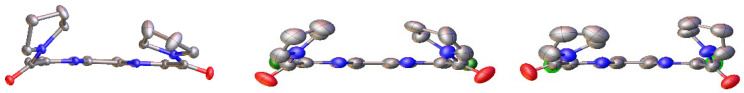
General view of ligands **L1** (**a**), **L2** (**b**), and **L3** (**c**) in two projections. The non-hydrogen atoms are shown as thermal ellipsoids at a 50% probability level [16,17].

As follows from the X-ray diffraction data (Figure 2), the carboxyl groups in all three **L1**–**L3** [15,17,23] ligands are on the same side of the phenanthroline core. The length of the C = O bonds varies from 1.220(3) Å in the ligand **L2** to 1.246(2) Å in the ligand **L1**. Some geometric parameters are given in Table 2.

**Figure 3:**
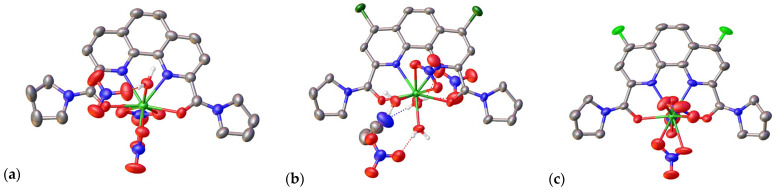

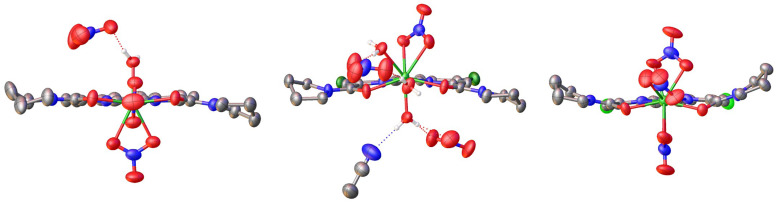
General view of the complexes **L1**•Lu(NO_3_)_3_ (**a**), **L2**•Lu(NO_3_)_3_ (**b**), and **L3**•Lu(NO_3_)_3_ (**c**) in two projections. Hydrogen atoms, except those of coordinated water molecules as well as the lattice solvent in **L3**•Lu(NO_3_)_3,_ are omitted for clarity, and the non-hydrogen atoms are shown as thermal ellipsoids at a 50% probability level.

Next, we synthesized complex compounds of ligands **L1**–**L3** with lutetium nitrate (see the Materials and Methods section and Supplementary Materials Figures S1–S6). In each of the cases, we were able to investigate the structure of the obtained complex compounds by combining various spectral methods (NMR and IR) and by XRD analysis (Figure 3). Table 3 shows the IR data of ligands **L1**–**L3** and their complexes with Lu(NO_3_)_3_. It can be seen from the data that moving from **L1** to **L3** results in the shift difference of C=O increasing to 20 cm^−1^.

Single crystals were obtained by slow isothermal recrystallization of these complexes from the MeCN/CHCl_3_ solvent system. The resulting crystals of **L2**•Lu(NO_3_)_3_ and **L3**•Lu(NO_3_)_3_ also contained a lattice acetonitrile molecule. In the complexes with the ligands **L1** and **L2**, the coordination number of the metal ion was nine (Figure 3).

In **L1**•Lu(NO_3_)_3_, the coordination environment of the metal ion includes the ligand **L1** and two nitrate anions acting as bidentate ligands (Table 4) while the third nitrate group is displaced by the water molecule. The O-H…O hydrogen bond (O…O 2.683(5) Å, OHO 170.2(2)°) holds the latter species together to produce a centrosymmetric dimer from the neighboring complex molecules (Figure 3). No stacking interactions occur despite the presence of the extended aromatic phenanthroline core in the ligand **L1**; apart from the above hydrogen bonds, only weak van der Waals interactions operate in the crystal of this complex.

**Figure 4:**
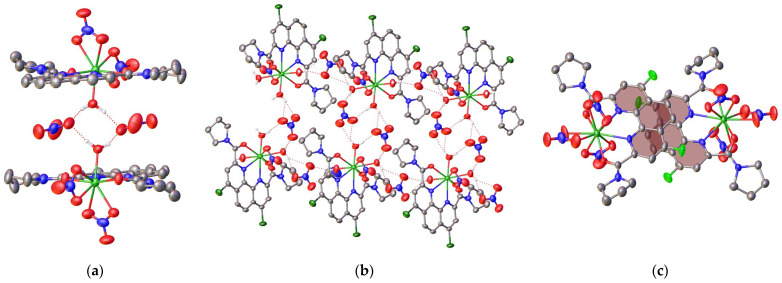
Fragments of the crystal packing illustrating the formation of a hydrogen-bonded dimer in **L1•**Lu(NO_3_)_3_ (**a**), a hydrogen-bonded tape in **L2•**Lu(NO_3_)_3_ (**b**), and a dimer formed by stacking interactions in **L3•**Lu(NO_3_)_3_ (**c**). Red dotted lines represent hydrogen bonds and the phenanthroline cores involved in stacking interactions are highlighted in pink.

In **L2•**Lu(NO_3_)_3_, two nitrate anions occur in the outer sphere so that the metal ion coordinates one nitrate anion in a similar bidentate manner and three water molecules (Figure 3). The latter three molecules are involved in hydrogen bonding with the outer-sphere nitrate anions (O…O 2.72(2)–3.264(6) Å, OHO 126.3(3)–175.7(3)°) and the lattice acetonitrile molecule (O…N 2.853(6) Å, OHN 177.5(3)°) to produce corrugated double tapes along the crystallographic axis *a* (Figure 4). These tapes are packed into the 3D framework by Cl…Cl halogen bonds [26], as judged by the Cl…Cl distance of 3.251(2) Å and the C-Cl…Cl angle of 166.97(13)°, and stacking interactions between the parallel phenanthroline cores (the interplane angle is 0° and the intercentroid and shift distances are 4.6063(18) and 3.065(3) Å, respectively).

The coordination environment of the metal ion in **L3•**Lu(NO_3_)_3_ is formed only by the ligand **L3** and three nitrate anions that all act as bidentate ligands. As a result, the coordination number reaches 10 (Figure 3). This type of coordination of the lutetium ion has already been observed in complexes with some organic ligands [27,28,29], however, it is still quite rare. For 1,10-phenanthroline-2,9-diamides, this is the first time that such a coordination number has been observed. As there are no convenient proton donors, such as the coordinated water molecules in **L1**•Lu(NO_3_)_3_ and **L2•**Lu(NO_3_)_3_, the crystal packing of **L3•**Lu(NO_3_)_3_ is dominated by parallel-displaced stacking interactions between the phenanthroline cores (the interplane angle is 0° and the intercentroid and shift distances are 4.217(5) and 2.507(8) Å, respectively) that produce centrosymmetric dimers (Figure 4).

In the complexes **L1•**Lu(NO_3_)_3_ and **L3•**Lu(NO_3_)_3_, the metal ion is located almost in the plane of the phenanthroline core of the ligand; the distance between the plane and the metal ion for these two complexes is 0.090(4) and 0.027(7) Å, respectively. In the complex **L2•**Lu(NO_3_)_3_, however, the metal ion moves away from the phenanthroline plane by 0.438(2) Å (Table 4). The Lu-N bond lengths increase in the row of substituents H–Cl–F in the four and seven positions of the ligands.

### 2.2. UV-Vis Titration

Stability constants for **L3** with four representatives of lanthanides La(III), Nd(III), Eu(III), and Lu(III) were determined using UV-vis spectrophotometry titration. The absorption band of **L3** in the range from 240 to 360 nm is very sensitive to its coordination environment. 

**Figure 5:**
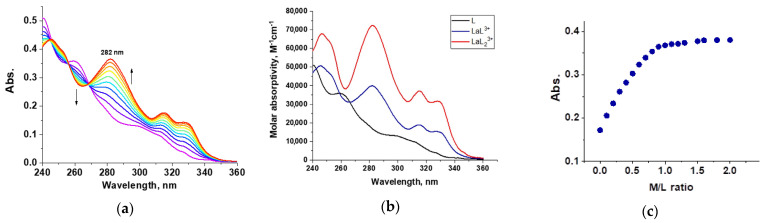
Spectrophotometric titration of **L3** (ca. 10^−5^ mol L^−1^) with La^3+^ ions (ca. 5 × 10^−4^ mol L^−1^) in CH_3_CN solution (T = 25.0 ± 0.1 °C, I = 0 M, V_0_ = 2.0 mL): (**a**) absorption spectra, (**b**) the molar absorptivities of free ligand **L3** and La(III) complexes calculated from spectral deconvolution, and (**c**) titration curve at maximum absorption (282 nm).

Thus, these spectral changes can be used to determine stability constants (log β). Figure 5 shows an example of the absorption spectrum obtained by spectrophotometric titration of the ligand **L3** with lanthanum trinitrate in a solution of dry acetonitrile (Figure 5a), the molar absorptions of free ligand **L3** and Eu(III) complexes calculated from spectral deconvolution (Figure 5b), and the titration curve at maximum absorption (Figure 5c). Similar data for Nd(III), Eu(III), and Lu(III) are shown in Supplementary Materials Figure S7. All spectra have similar trends of change. With an increase in the number of metal ions added, the peak of the ligand **L3** (~260 nm) gradually decreases, and a new peak corresponding to the metal-ligand complex appears in the 280 nm region. The obtained titration curves were analyzed using the Hypspec2014 program [30]. For all studied lanthanide ions, it was found that the titration data best correspond to the formation of **L3**:metal complexes 1:1 and 2:1 stoichiometry, which was previously also observed for **L1** and **L2** ligands [16,17]. The obtained stability constants of complexes of **L3** with lanthanide ions (log β) are shown in Table 5. The **L3** ligand has a greater affinity for light lanthanides since a decrease in log β1 is observed during the transition from La to Lu. It is worth noting that the stability constants for the **L3** ligand with La^3+^, Nd^3+^, and Eu^3+^ are approximately 0.5 greater than the stability constants for the **L1** and **L2** ligands, while the log β1 values for complexes with Lu^3+^ are similar for all three ligands (log β1 = 5.98 ± 0.02 for **L1** and 6.06 ± 0.02 for **L2**). The reason for this has been discussed above.

### 2.3. NMR Titration

**Figure 6:**
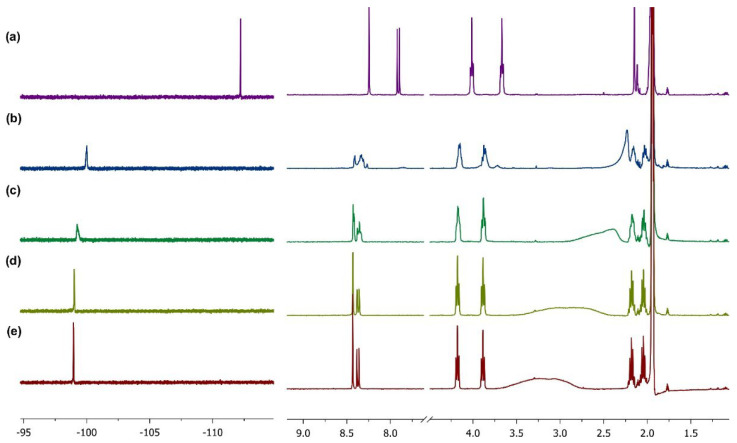
^19^F (left) and ^1^H (right) spectra of NMR titration in CD_3_CN at 25 °C. From top to bottom (**a**) before adding Lu^3+^, (**b**) **L3**: Lu^3+^ = 1:0.5, (**c**) **L3**: Lu^3+^ = 1:1, (**d**) **L3**: Lu^3+^ = 1:1.5, and (**e**) **L3**: Lu^3+^ = 1:2.

Next, we studied the complexation of **L3** with lutetium trinitrate at 25 °C using ^1^H and ^19^F NMR titration in deuterated acetonitrile (Figure 6). The gradual addition of a solution of lutetium pentahydrate Lu(H_2_O)_5_(NO_3_)_3_ in CD_3_CN to a solution of **L3** in the same solvent led to a downfield shift of phenanthroline H^5,6^ and H^3,8^ signals as well as signals of the α-CH_2_ groups of the pyrrolidine moieties. Adding two equimoles of lutetium trinitrate caused the H^5,6^ and H^3,8^ signals to shift by 0.18 and 0.46 ppm, respectively, and the signals of the α-CH_2_ groups to shift by 0.17 and 0.22 ppm. An especially significant shift was observed in the ^19^F NMR. For example, the starting ligand had a signal with a shift of −112.22 ppm, which decreased to −99.25 ppm and −98.95 ppm after the addition of one or two equimoles of lutetium trinitrate, respectively. At the same time, the spectrum continued to change slightly even with the addition of lutetium nitrate to the solution of the 1:1 stoichiometry complex. In the case of stoichiometry, clear signals are observed in both the ^1^H and ^19^F NMR spectra when the metal:ligand ratio is 2:1, which may indicate that in the case of 1:1 stoichiometry, the ligand molecules may not be equivalent [31]. 

**Figure 7:**
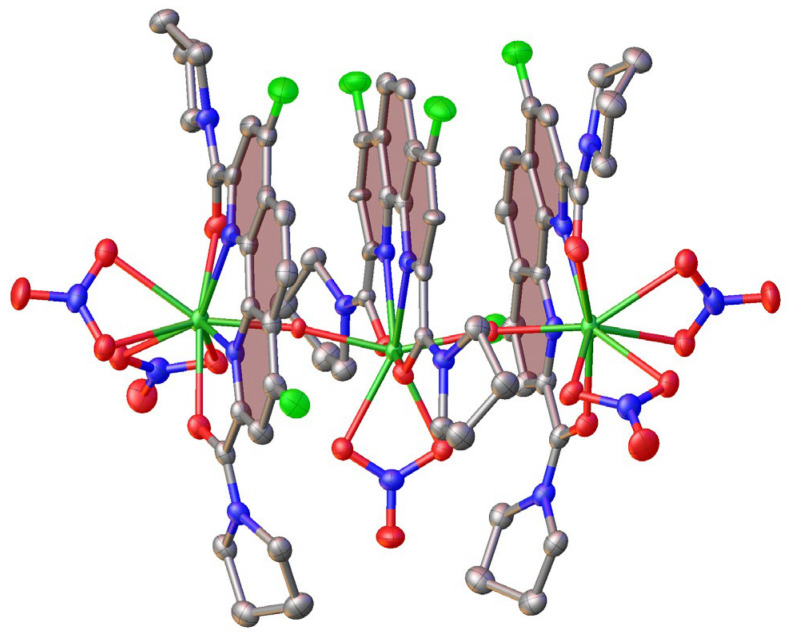
General view of the oxo-complex (**L3**)**_3_**Lu_3_O_2_(NO_3_)_5_ with hydrogen atoms omitted for clarity and other atoms shown as thermal ellipsoids at a 50% probability level. Phenanthroline cores involved in stacking interaction are highlighted by pink.

Having identified the structural differences in the studied lutetium complexes, the question of whether the ligands **L2** and **L3** are able to produce complexes with a metal:ligand ratio of 1:2 arose. We attempted to synthesize such a complex by reacting two ligand equivalents with lutetium nitrate. With the **L2** ligand, we again obtained a complex with a metal:ligand raio of 1:1. With the ligand **L3,** a poly-nuclear oxo-complex (**L3)_3_**Lu_3_O_2_(NO_3_)_5_ in which the monomers are interconnected via the Lu-O bond, was obtained (Figure 7). In this complex, there are two symmetry-independent metal ions, the one in the center—Lu(2)—with a coordination number of eight and the two at the periphery—Lu(1)—with a coordination number of nine. The coordination environment of the former is formed by the ligand **L3**, with one nitrate anion coordinated in a bidentate manner and two oxygen atoms in the axial positions (Table 6). The latter coordinate involves another nitrate anion instead of one of the oxygen atoms. The central metal ion is located almost in the plane of the phenanthroline core of the ligand **L3** (the distance from the plane and the metal ion is only 0.053(15) Å), while the metal ion at the periphery is shifted by 0.207(4) Å from this plane away from the central metal ion (Table 6).

In addition to the above coordinate bonds, the complex (**L3**)**_3_**Lu_3_O_2_(NO_3_)_5_ is stabilized by intramolecular parallel-displaced stacking interactions between the phenanthroline cores; the appropriate interplane angles are 5.27(14) and 6.0(2)°, the intercentroid distances are 4.476(4) and 3.662(5) Å, and the shift distances are 2.663(4) and 2.974(6) Å. Therefore, only weak van der Waals interactions pack the complex molecules into the 3D framework. 

### 2.4. Extraction of Am(III) and Eu(III)

**Figure 8:**
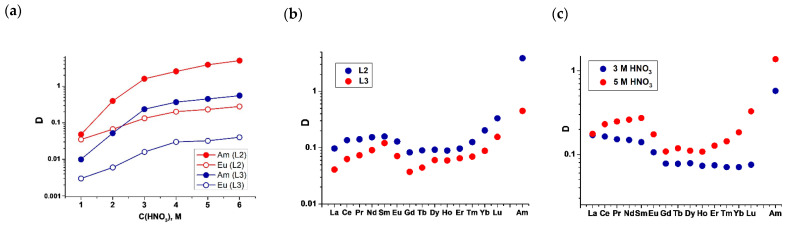
The dependence of the distribution ratio Am(III) and Eu(III) on the concentration of nitric acid in the equilibrium aqueous phase during extraction with 0.01 M ligand solutions in F3 (**a**). Distribution ratios of lanthanides(III) and Am(III) for extraction by (**b**) 0.01 mol L^−1^ **L2** and **L3** from 5 mol L^−1^ HNO_3_ and (**c**) 0.05 mol L^−1^ **L3** from 3 and 5 mol L^−1^ HNO_3_.

For ligands **L2** and **L3**, the distribution ratio and separation factors of the Am(III)/Eu(III) pair were determined during extraction from nitric acid solutions with 0.01 M ligand solutions in 3-nitrobenzotrifluoride (F3) (Figure 8, Table 7). Ligand **L1** did not participate in extraction experiments since this ligand has a negative log P = −0.08 and, as we have shown earlier, the distribution coefficients of Am(III) and Eu(III) during extraction with this ligand decrease with increasing phase contact time, which may be due to the fact that the complexes transition to the aqueous phase. The introduction of electron σ-acceptor fluorine atoms, which are stronger than chlorine atoms, leads to a greater decrease in the electron density on phenanthroline nitrogen atoms. As a result, the distribution ratios of Am(III) and Eu(III) are 5–10 times smaller in the case of extraction with **L3** compared with **L2**.

However, the introduction of electron σ-acceptor atoms (both chlorine and fluorine) into the positions four and seven of the phenanthroline fragment leads to a decrease in the Brønsted basicity of diamides for ligands **L2** and **L3**. As a result, an increase in the distribution coefficients of Am(III) and Eu(III) is observed over the entire studied range of nitric acid concentrations (1–6 mol·L^−1^). Unsubstituted diamides usually have an extraction maximum at a nitric acid concentration of 3–4 mol·L^−1^. A higher acid content leads to ligand protonation and a decrease in the distribution coefficients [17,32].

The extraction of lanthanides(III) (except for Pm(III)) was also studied. Figure 8b shows the distribution coefficients of the lanthanides(III) in the extraction of 0.01 M **L2** and **L3** from 5 M HNO_3_. The distribution coefficients of lanthanides(III) also take smaller values in the case of extraction with diamide with more σ-electron-withdrawing fluorine atoms, like what is seen with **L3**. Since the distribution coefficients of lanthanides(III) and americium(III) in the extraction of **L3** under these conditions take values less than one, we also studied the extraction of lanthanides(III) with 0.05 M solutions of **L3** in F3 from 3 and 5 M HNO_3_. During extraction from 5 M HNO_3_, the distribution coefficients of lanthanides(III) also turned out to be less than one, while the distribution coefficient of Am(III) was greater than one, which made it possible to separate it from lanthanides(III). It should be noted that in all cases, Am(III) was extracted better than lanthanides(III). The dependence of the distribution coefficients D on the cation atomic number Z when moving along the lanthanide series has a V-shaped character, which has already been observed for ligands of this type. The reasons for the appearance of such dependencies were considered by us earlier [14]. 

## 3. Materials and Methods

Chemical reagents such as Lu(NO_3_)_3_·xH_2_O and other inorganic/organic reagents and solvents were of analytical grade. Lutetium trinitrate hydrate Lu(NO_3_)_3_·xH_2_O was purchased from Sigma-Aldrich, Co. (St. Louis, MO, USA) and used without further purification. The water content x in lutetium nitrate was determined as x = 5. Deuterated solvent CD_3_CN for NMR spectra registration was purchased from Cambridge Isotope Laboratories, Inc. (Andover, MA, USA) and used without further purification. Acetonitrile, chloroform, and diethyl ether which were used for the synthesis of complexes were purified according to known procedures. C_6_F_6_ was purchased from Sigma-Aldrich, Co. (St. Louis, MO, USA) and used without further purification. Analytical grade 3-nitrobenzotrifluoride (F3) was purchased from Rhodia (France).

NMR spectra were recorded using standard 5 mm sample tubes on an Agilent 400-MR spectrometer (Agilent Technologies, Santa Clara, CA, USA) with operating frequencies of 400.1 MHz (^1^H) and 376 MHz (^19^F). IR spectra in the solid state were recorded on a Nicolet iS5 FTIR spectrometer (Thermo Fisher Scientific, Waltham, MA, USA) using an internal reflectance attachment with a diamond optical element and an attenuated total reflection (ATR) with a 45° angle of incidence. The resolution was 4 cm^−1^ and the number of scans was 32. HRMS ESI mass spectra were recorded on the MicroTof Bruker Daltonics and Orbitrap Elite instruments.

All quantum chemical calculations were performed by the Gaussian 16 program [25] DFT method with B3LYP functional and basis 6-31G (d,p).

### 3.1. Synthesis and Analytical Data

Syntheses of ligands **L1**, **L2** and **L3** were reported earlier [15,17,23].

General procedure for preparation of the complexes with Lu(NO_3_)_3_.

A solution of lutetium nitrate (0.1 mmol) in acetonitrile (1 mL) was added dropwise to a solution of 1,10-phenanthroline-2,9-dicarboxamide (0.1 mmol) in chloroform (1 mL) . After, the reaction mixture was concentrated in a vacuum to 1/10 of its initial volume and then treated with diethyl ether (2 mL). The resulting complex was filtered and washed with ether and dried in air.

N^2^,N^9^-bis(pyrrolidine)-N^2^,N^9^-diethyl-1,10-phenanthroline-2,9-dicarboxamide lutetium trinitrate **L1**•Lu(NO_3_)_3_. Yield 88.7% (65.2 mg). White powder. T_decomp._ > 340 °C; ^1^H NMR (CD_3_CN) δ 8.93 (d, *J* = 8.6 Hz, 2H, Phen), 8.59 (d, *J* = 8.6 Hz, 2H, Phen), 8.29 (s, 2H, Phen), 4.19 (t, *J* = 6.9 Hz, 4H, Pyrr), 3.89 (t, *J* = 6.9 Hz, 4H, Pyrr), 2.21–2.14 (m, 4H, Pyrr) 2.08–2.01 (m, 4H, Pyrr); IR (ν, cm^−1^) 3149, 3069, 2975, 2878 (C-H stretching vibrations), 1602 (C=O); HRMS (ESI-TOF) (m/z) [M + H^+^] calculated for [C_22_H_22_LuN_6_O_8_]^+^ 673.0902, found 673.0886.

N^2^,N^9^-bis(pyrrolidine)-4,7-dichloro-N^2^,N^9^-diethyl-1,10-phenanthroline-2,9-dicarboxamide lutetium trinitrate **L2**•Lu(NO_3_)_3_. Yield 88.1% (70.5 mg). Yellow powder. T_decomp._ 240 °C; ^1^H NMR (CD_3_CN) δ 8.62 (s, 2H, Phen), 8.58 (s, 2H, Phen), 4.18 (t, *J* = 6.9 Hz, 4H, Pyrr), 3.88 (t, *J* = 6.9 Hz, 4H, Pyrr), 2.20–2.13 (m, 4H, Pyrr), 2.07–2.00 (m, 4H, Pyrr); IR (ν, cm^−1^) 3120, 3083, 2987, 2883 (C-H stretching vibrations), 1609 (C=O); HRMS (ESI-TOF) (*m*/*z*) [M + H^+^] calculated for [C_22_H_20_Cl_2_LuN_6_O_8_]^+^ 741.0122, found 741.0122 

N^2^,N^9^-bis(pyrrolidine)-4,7-difluoro-N^2^,N^9^-diethyl-1,10-phenanthroline-2,9-dicarboxamide lutetium trinitrate **L3**•Lu(NO_3_)_3_. Yield 96.8% (74.6 mg). White powder. T_decomp._ 250 °C; ^1^H NMR (CD_3_CN) δ 8.40 (s, 2H, Phen), 8.33 (d, *J* = 9.8 Hz, 2H, Phen), 4.16 (t, *J* = 6.9 Hz, 4H, Pyrr), 3.87 (t, *J* = 6.9 Hz, 4H, Pyrr), 2.21–2.12 (m, 4H, Pyrr), 2.07–1.99 (m, 4H, Pyrr); ^19^F NMR (376 MHz, Acetonitrile-*d*_3_) δ −99.52 (d, *J* = 9.8 Hz), −103.28; IR (ν, cm^−1^) 3115, 3087, 2979, 2881 (C-H stretching vibrations), 1608 (C=O); HRMS (ESI-TOF) (*m*/*z*) [M + H^+^] caclulated for [C_22_H_20_F_2_LuN_6_O_8_]^+^ 709.0713, found 709.0705

Oxo-complex (**L3**)**_3_**Lu_3_O_2_(NO_3_)_5_ was obtained in accordance with the general procedure starting from 0.2 mmol of **L3** and 0.1 mmol of lutetium nitrate. The treatment of the residue with ether yielded 112.8 mg of yellowish powder. This material was used for growing single crystals.

### 3.2. UV-Vis Titration Experiment

UV−VIS spectra were recorded at a temperature of 25.0 ± 0.1 °C in the wavelength region of 200−500 nm (0.5 nm interval) on a Shimadzu UV 1800 spectrophotometer controlled by LabSolutions version 1.2.35 UV-Vis software with a thermostatic attachment (Shimadzu TCC-100) using quartz cuvettes with an optical path length of 10 mm. A stock solution of the ligand was prepared (ca. 10^−4^ mol L^−1^) by dissolving the respective ligand in CH_3_CN; a working ligand solution (ca. 10^−5^ mol L^−1^) was then prepared from the initial solution. A working titrant solution (10^−3^ mol L^−1^) was prepared by dissolving the respective lanthanide(III) nitrate hydrate Ln(NO_3_)_3_·xH_2_O compound (Ln = La, Nd, Eu, Lu; x = 6 in the case of La, Nd, Eu and x = 5 in the case of Lu) in CH_3_CN. Acetonitrile (CH_3_CN; 99.95%, HPLC grade, Panreac AppliChem) was dried over molecular sieves (zeolite KA, 3 Å, balls, diameter 1.6−2.5 mm, production HKC Corp., Hong Kong) prior to use. The titration was carried out by adding 2 μL aliquots of the working metal cation solution to 2 mL of the working ligand solution in the titration cell. The titration continued until no obvious change was observed in the spectra. The stability constants of the Ln(III) complexes were calculated using the HypSpec2014 program.

### 3.3. NMR Titration Experiment

For the NMR titration experiments, the stock solutions of **L3** and Lu(NO_3_)_3_ were obtained by dissolving weighed amounts of ligand **L3** and Lu(NO_3_)_3_·5H_2_O in CD_3_CN, respectively. The initial aliquot solution of **L3** was divided equally into five NMR tubes equally and a certain amount of Lu(NO_3_)_3_ solution was added to four of them to obtain a series of samples with molar ligand:metal ratios of 1:0, 1:0.5, 1:1, 1:1.5, and 1:2. The total concentrations of metals and ligands were 0.0585 mol L^−1^ and 0.0244 mol L^−1^ , respectively. After the addition, ^1^H and ^19^F NMR spectra were recorded on an Agilent 400-MR instrument with a frequency of 400.1 MHz (^1^H) and 376 MHz (^19^F). C_6_F_6_ in CD_3_CN was used as a reference for the ^19^F-NMR spectra.

### 3.4. X-ray Crystallography

X-ray diffraction data for the ligand **L3** and for the complexes **L•**Lu(NO_3_)_3_ were collected at 295 K using a STOE STADIVARI diffractometer with a Pilatus 100K detector using graphite monochromated Mo-Kα radiation (λ = 0.71073 Å) for **L2•**Lu(NO_3_)_3_ and the Cu Kα radiation (λ = 1.54186 Å) from the fine-focus Cu GeniX 3D radiation source for the others. The data for (**L3)_3_**Lu_3_O_2_(NO_3_)_5_ were collected at 100 K with a Bruker Quest D8 CMOS diffractometer using graphite monochromated Mo-Kα radiation (λ = 0.71073 Å). The structures of the ligand **L3** and of the complexes **L•**Lu(NO_3_)_3_ were solved and refined with the program SHELX [33], while the ShelXT [34] structure solution program using intrinsic phasing and refined with the XL [33] refinement package using least-squares minimization against F^2^ was used for (**L3)_3_**Lu_3_O_2_(NO_3_)_5_. The non-hydrogen atoms were refined by using the anisotropic full matrix least-squares procedure. Hydrogen atoms of coordinated water molecules were located from different Fourier syntheses while the positions of others were calculated, and all of them were refined in isotropic approximation within the riding model. Crystal data and structure refinement parameters for the ligand **L3** and the complexes **L•**Lu(NO_3_)_3_ and (**L3)_3_**Lu_3_O_2_(NO_3_)_5_ are given in Table S1 (see the Supplementary Materials). 

CCDC 2221666 (for **L3**), 2221667 (for **L1•**Lu(NO_3_)_3_), 2191901 (for **L2•**Lu(NO_3_)_3_), 2221670 (for **L3•**Lu(NO_3_)_3_), and 2232270 (for (**L3)_3_**Lu_3_O_2_(NO_3_)_5_) contain the supplementary crystallographic data for this paper. 

## 4. Conclusions

In summary, we revealed important structural features of complexes of lutetium trinitrate with pyrrolidine-derived phenanthroline diamides **L1**–**L3**. Depending on the presence and type of halide atoms in positions four and seven of the phenanthroline core, the coordination number of lutetium can be equal to either nine (complexes of **L1** and **L2**), which is typical for lutetium complex compounds, or 10 (fluorine-containing ligand **L3**), which is very rare. The measurement of bond lengths in the structures of complexes studied by the XRD analysis shows that in the case of **L3**, the bonds of the lutetium atom with the coordination centers are longer than similar bonds in the complexes of the other two ligands. Also, in the case of the lutetium trinitrate complex with **L3**, the smallest out-of-plane distance of the metal from the phenanthroline plane is observed. Apparently, this makes the coordination of three bidentate nitrate groups possible. It is also noteworthy that in the case of a complex with the **L2** ligand, only one of three nitrate groups is located in the internal coordination sphere, while the other two nitrate groups are displaced by three compact water molecules. 

Using UV-Vis and NMR titration techniques, we elucidated the coordination chemistry of ligands in acetonitrile solutions and obtained the values of stability constants. The log β2 values for lanthanide complexes of **L3** turned out to be much higher than those of the corresponding complexes of ligands **L1** and **L2**. Counting on greater stability of the **L3**:lanthanide = 2:1 complexes, we tried to obtain complexes of such stoichiometry with lutetium trinitrate in an individual form. As a result, the oxo-complex was isolated using XRD analysis. It is very likely that in this case, one ligand molecule works as a base by binding the nitrate anion.

In addition, we performed preliminary liquid–liquid extraction tests. The results obtained indicate a lower extraction efficiency of the ligand **L3** compared to its chlorine-containing analog **L2**, but the selectivity in the separation of the Am(III)/Eu(III) remains high. This is probably the result of the lower lipophilicity of the **L3** ligand, which affects the formation of water-soluble complexes with lanthanide nitrates.

## Figures and Tables

**Table 1:** ESP charges of N_Phen_ and O_amide_ atoms of ligands **L1**–**L3**.

Ligand	ESP Charges	
	N_Phen_	O_amide_
**L1**	−0.339/−0.367	−0.500/−0.502
**L2**	−0.263/−0.307	−0.488/−0.489
**L3**	−0.366/−0.405	−0.501/−0.502

**Table 2:** Some geometric parameters of ligands **L1**–**L3** [16,17].

Ligand/Bond Length, Å	C = O(1)	C = O(2)	C-N(Amide)	C-N(Amide)	OCCN(°)	OCCN(°)
**L1**	1.241(2) [1.246(2)]	1.241(2) [1.245(2)]	1.347(2) [1.337(2)]	1.348(2) [1.344(2)]	139.48(16) [150.64(18)]	160.72(16) [173.73(18)]
**L2**	1.220(3)	1.223(3)	1.328(3)	1.332(3)	128.6(3)	141.1(3)
**L3**	1.232(4)	1.235(4)	1.330(4)	1.324(4)	124.6(3)	152.6(3)

The values for the second symmetry-independent molecule are given in brackets.

**Table 3:** IR data of ligands **L1**–**L3** and their complexes with Lu(NO_3_)_3_.

	IR (CO), cm^−1^		IR (CO), cm^−1^	Δ (CO), cm^−1^
**L1**	1613	**L1**•Lu(NO_3_)_3_	1602	11
**L2**	1622	**L2**•Lu(NO_3_)_3_	1609	13
**L3**	1628	**L3**•Lu(NO_3_)_3_	1608	20

**Table 4:** Some geometric parameters of the complexes **L**•Lu(NO_3_)_3_.

	L1•Lu(NO_3_)_3_	L2•Lu(NO_3_)_3_	L3•Lu(NO_3_)_3_
Bond Length, Å			
R_M-O(1)_	2.304(3)	2.311(2)	2.324(5)
R_M-O(2)_	2.267(3)	2.321(3)	2.313(5)
R_M-N(1)_	2.416(3)	2.472(3)	2.500(6)
R_M-N(2)_	2.404(3)	2.483(3)	2.531(6)
R_M-ONO2(2)_	2.363(3)–2.471(4)	2.405(3), 2.478(3)	2.406(7)–2.490(6)
R_M-OH2_	2.281(3)	2.260(2)–2.392(3)	-
Out-of-plane	0.090(4)	0.438(2)	0.027(7)
Torsions (°)			
N_phen_-C-C=O	1.8(6), 11.5(6)	4.6(4), 6.0(4)	24.4(11), 17.4(12)
C_amide1_-N_amide_-C=O ^a)^	173.2(5), 177.3(5)	173.6(3), 167.3(5) [173.4(12)]	172.0(8), 174.0(10)
C_amide2_-N_amide_-C=O ^a)^	0.6(7), 2.7(5)	1.8(5), 6.2(6) [16.8(8)]	8.5(13), 1.4(14)

^a)^ The values for the minor component of the disordered moiety are given in brackets.

**Table 5:** Stability constants (log β) for **L1**, **L2**, and **L3** complexes with Ln(III) nitrates.

Ligand	Metal Ion	log β1	log β2
**L1**	La^3+^	5.90 ± 0.02	11.64 ± 0.04
	Nd^3+^	5.96 ± 0.02	11.78 ± 0.04
	Eu^3+^	5.92 ± 0.02	11.62 ± 0.04
	Lu^3+^	5.98 ± 0.03	11.78 ± 0.04
**L2**	La^3+^	5.82 ± 0.02	11.63 ± 0.05
	Nd^3+^	5.85 ± 0.02	11.64 ± 0.05
	Eu^3+^	5.90 ± 0.02	11.66 ± 0.05
	Lu^3+^	6.06 ± 0.02	11.63 ± 0.04
**L3**	La^3+^	6.53 ± 0.02	11.81 ± 0.06
	Nd^3+^	6.50 ± 0.01	11.70 ± 0.03
	Eu^3+^	6.39 ± 0.02	11.62 ± 0.04
	Lu^3+^	6.02 ± 0.02	12.04 ± 0.04

**Table 6:** Some geometric parameters of the oxo-complex (**L3**)**_3_**Lu_3_O_2_(NO_3_)_5_ ^a)^.

	Lu(1)	Lu(2)
Bond length, Å		
R_M-O(1),_ R_M-O(2)_	2.311(4), 2.320(4)	2.34(2) [2.24(2)]
R_M-N(1),_ R_M-N(2)_	2.463(4), 2.420(4)	2.431(7) [2.400(11)]
R_M-ONO2(2)_	2.390(18)–2.474(4)	2.375(3)
R_M-O_	2.129(2)	2.116(2)
Out-of-plane shift	0.207(4)	0.053(15)
Bond angles (°)		
Lu-O-Lu	170.99(14)	
O-Lu-O	-	154.79(14)
Torsions (^o^)		
N_phen_-C-C=O	12.1(6), 13.3(6)	9.6(18) [10(2)]
C_amide_-N_amide_-C=O	2.7(7)–3(4)	1(2) [2(2)]

^a)^ The values for the minor component of the disordered moiety are given in brackets.

**Table 7:** The values of the Am/Eu pair separation factors during extraction with 0.01 M ligand solutions in F3 from nitric acid solutions.

C(HNO_3_), M	L2	L3
1	1.4	3.3
2	5.9	8.7
3	12.1	14.8
4	12.7	12.3
5	16.7	14.1
6	18.0	13.9

## Data Availability

Samples of the compounds are not available from the authors.

## Supplementary Materials

The following supporting information can be downloaded at: https://www.mdpi.com/article/10.3390/ijms24065569/s1.
