# Supplementary material for: Pyrrolidine-Derived Phenanthroline Diamides: An Influence of Fluorine Atoms on the Coordination of Lu(III) and Some Other f-Elements and Their Solvent Extraction
Source: Int J Mol Sci. 2023 Mar 14;24(6):5569. doi: 10.3390/ijms24065569 (PMC10056393; doi:10.3390/ijms24065569)
Supplement: Supplementary file 1 [file ijms-24-05569-s001.zip › ijms-2265955-supplementary.pdf]

Electronic Supplementary Information

Pyrrolidine derived phenanthroline diamides: an influence of fluorine atoms on the coordination of Lu(III) and some other f-elements and their solvent extraction

Nane A. Avagyan,[1] Pavel S. Lemport,[1] Mariia V. Evsiunina,[1] Petr I. Matveev,[1] Svetlana A. Aksenova,[2,3] Yulia V. Nelyubina,[2] Alexandr V. Yatsenko,[1] Viktor A. Tafeenko,[1] Vladimir G. Petrov,[1] Yuri A. Ustynyuk,[1] Xihe Bi, [4] Valentine G. Nenajdenko[1]*

[1] Department of Chemistry, Lomonosov Moscow State University, Leninskie gory 1 bld. 3, Moscow, Russia; [2] A.N. Nesmeyanov Institute of Organoelement Compounds, Russian academy of Sciences, 119334, Moscow, Russia
[3] Moscow Institute of Physics and Technology, National Research University, Institutskiy per. 9, Dolgoprudny, 141700 Moscow Region, Russia
[4] Department of Chemistry, Northeast Normal University, 5286 Renmin Street, 130024, Changchun, China
* Correspondence to: nenajdenko@gmail.com

Table of contents

1. NMR and IR spectra of synthesized compounds……………………………………S2-S7
2. X-ray analysis data ……………………………………………………………..……..S8
3. Spectrophotometric titration……………………………………………………….…S9

1. NMR and IR spectra of synthesized compounds

N²,N⁹-bis(pyrrolidine)-N²,N⁹-diethyl-1,10-phenanthroline-2,9-dicarboxamide lutetium trinitrate L1*Lu(NO₃)₃

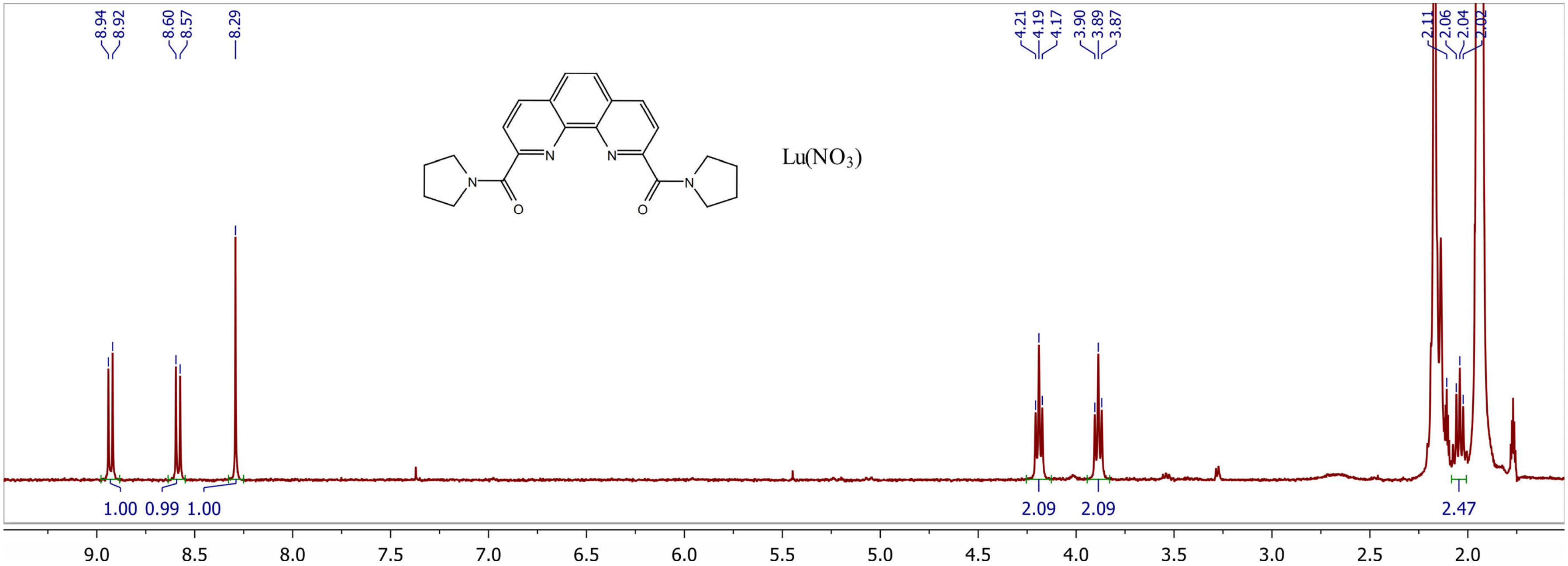

Figure S1. ^{1}H NMR spectrum of **L1*Lu(NO₃)₃** in CD_3CN at 25ºC

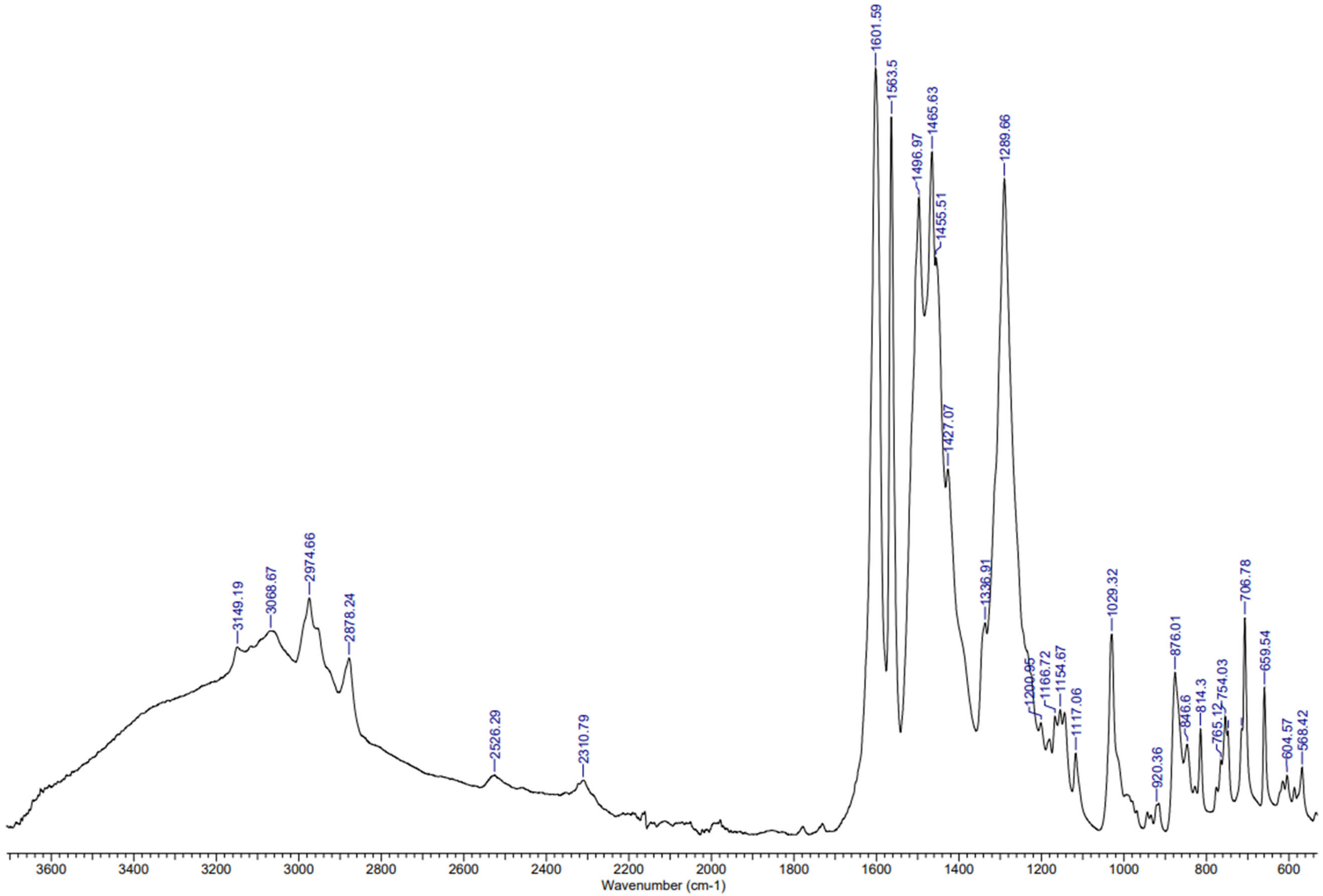

Figure S2. Solid-state IR of **L1*Lu(NO$_3$)$_3$** spectrum at 25ºC

N²,N⁹-bis(pyrrolidine)-4,7-dichloro-N²,N⁹-diethyl-1,10-phenanthroline-2,9-dicarboxamide lutetium trinitrate L2*Lu(NO_3)₃

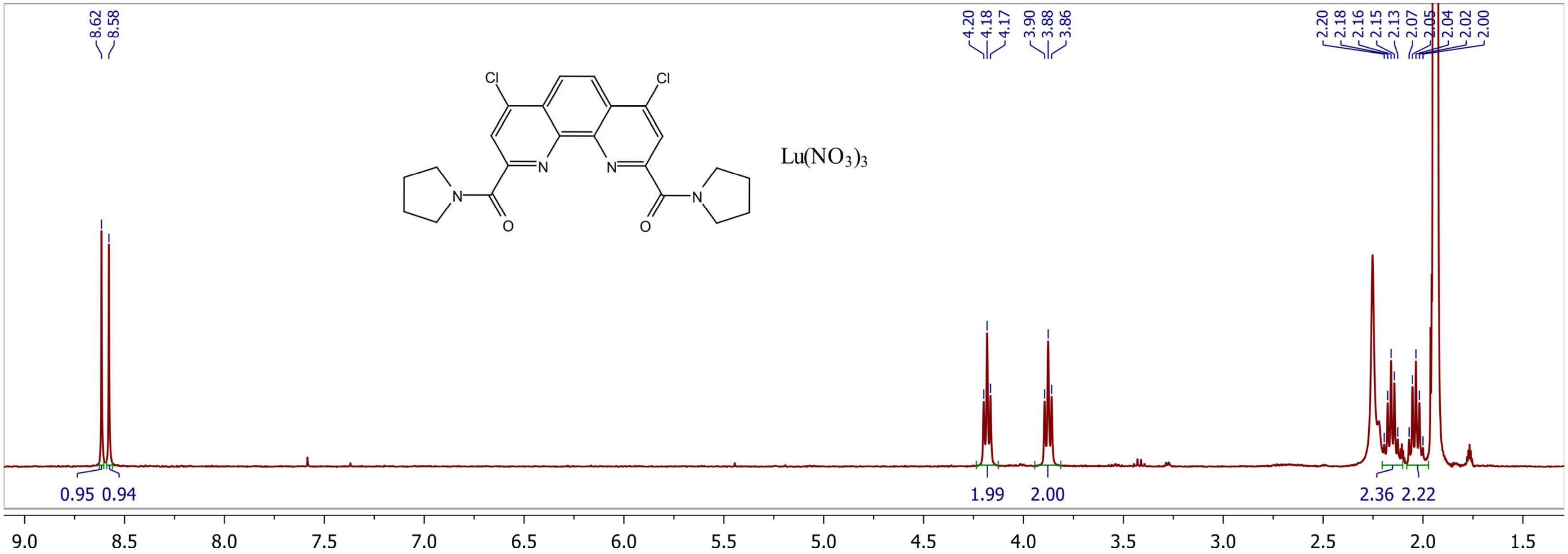

Figure S3. ¹H NMR spectrum of **L2*Lu(NO_3)₃** in CD_3CN at 25ºC

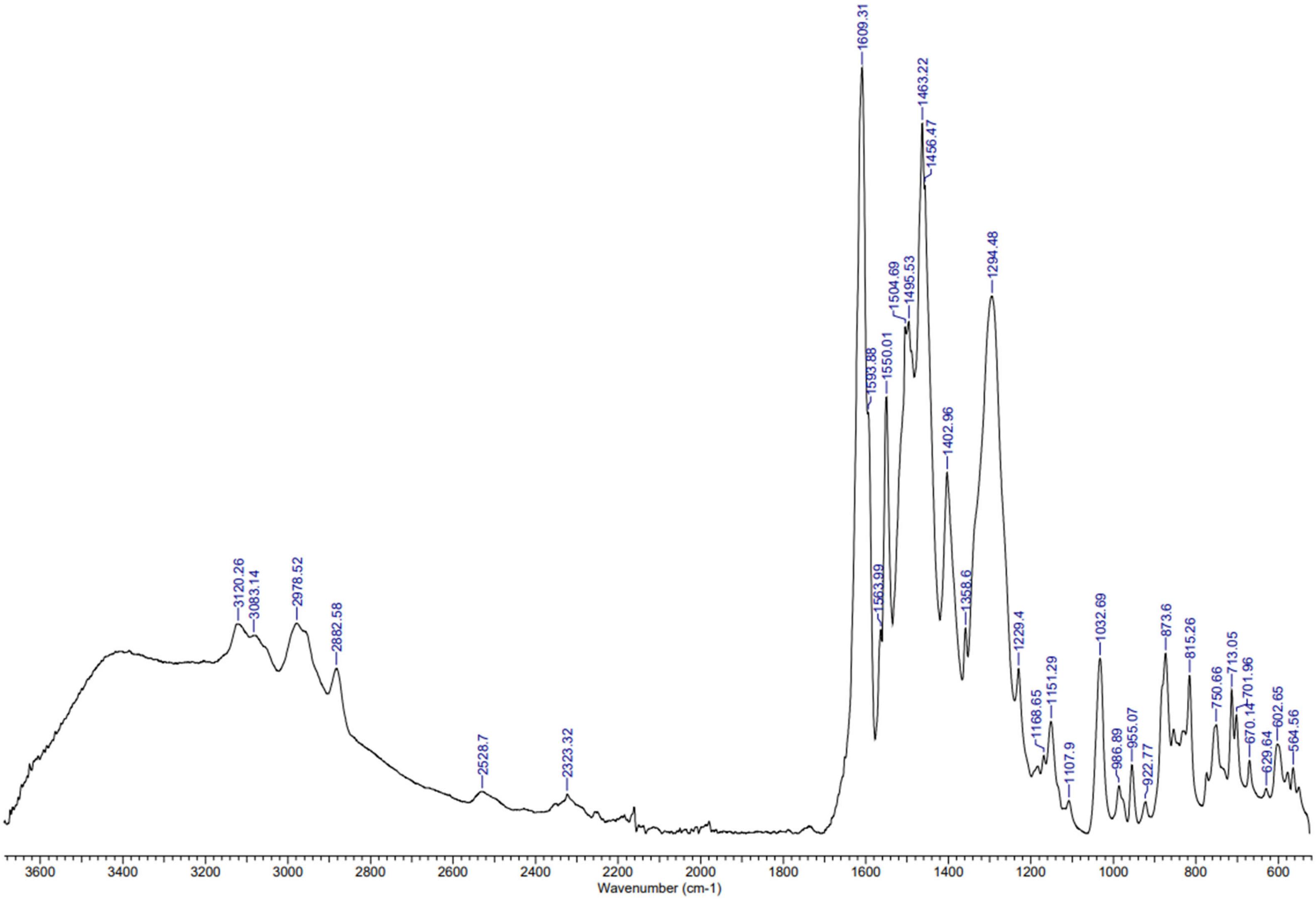

Figure S4. Solid-state IR spectrum of **L2*Lu(NO$_3$)$_3$** at 25ºC

N²,N⁹-bis(pyrrolidine)-4,7-difluoro-N²,N⁹-diethyl-1,10-phenanthroline-2,9-dicarboxamide lutetium trinitrate L3*Lu(NO₃)₃

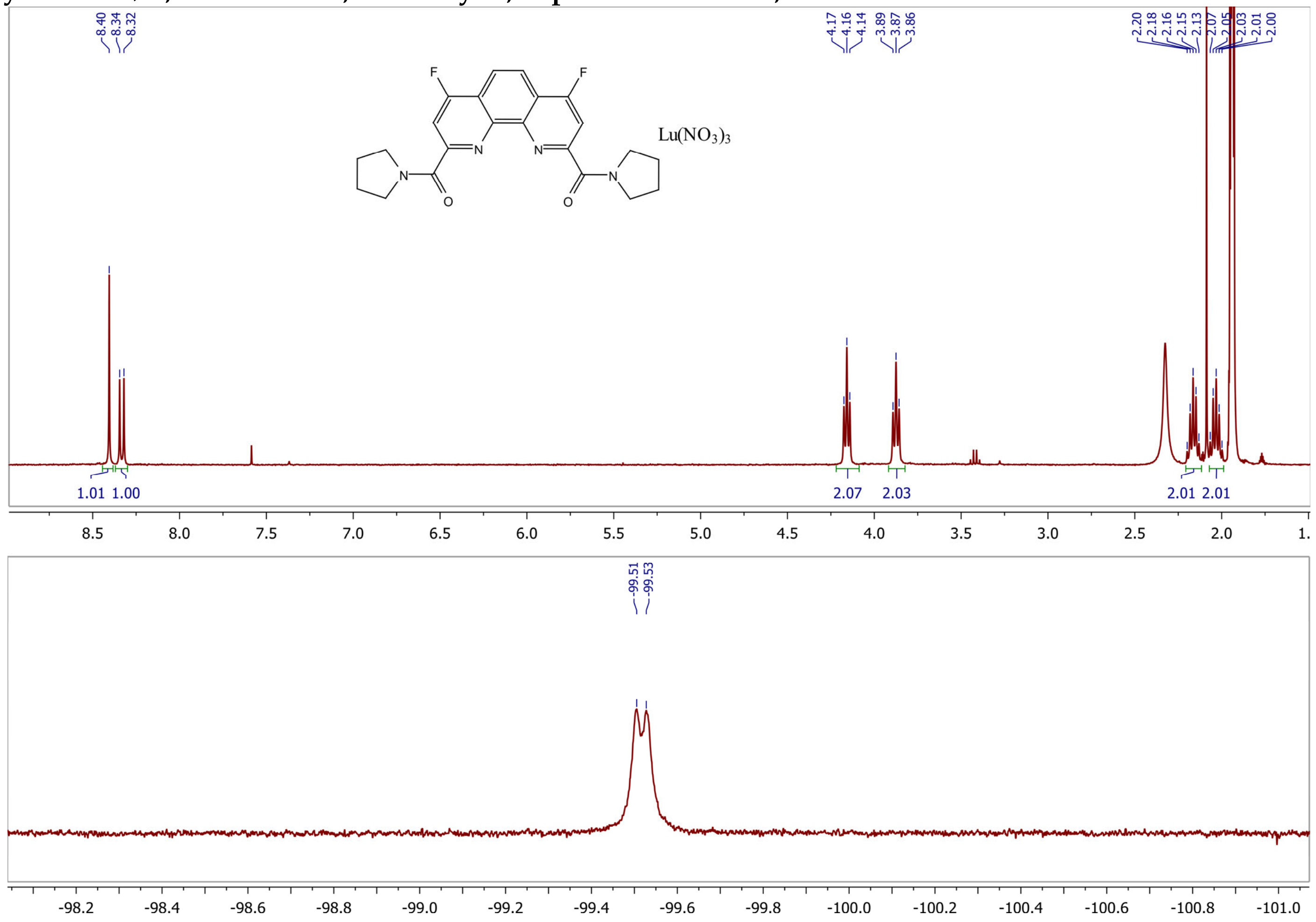

Figure S5. ^{1}H and ^{19}F NMR spectra of **L3*Lu(NO₃)₃** in CD_3CN at 25ºC

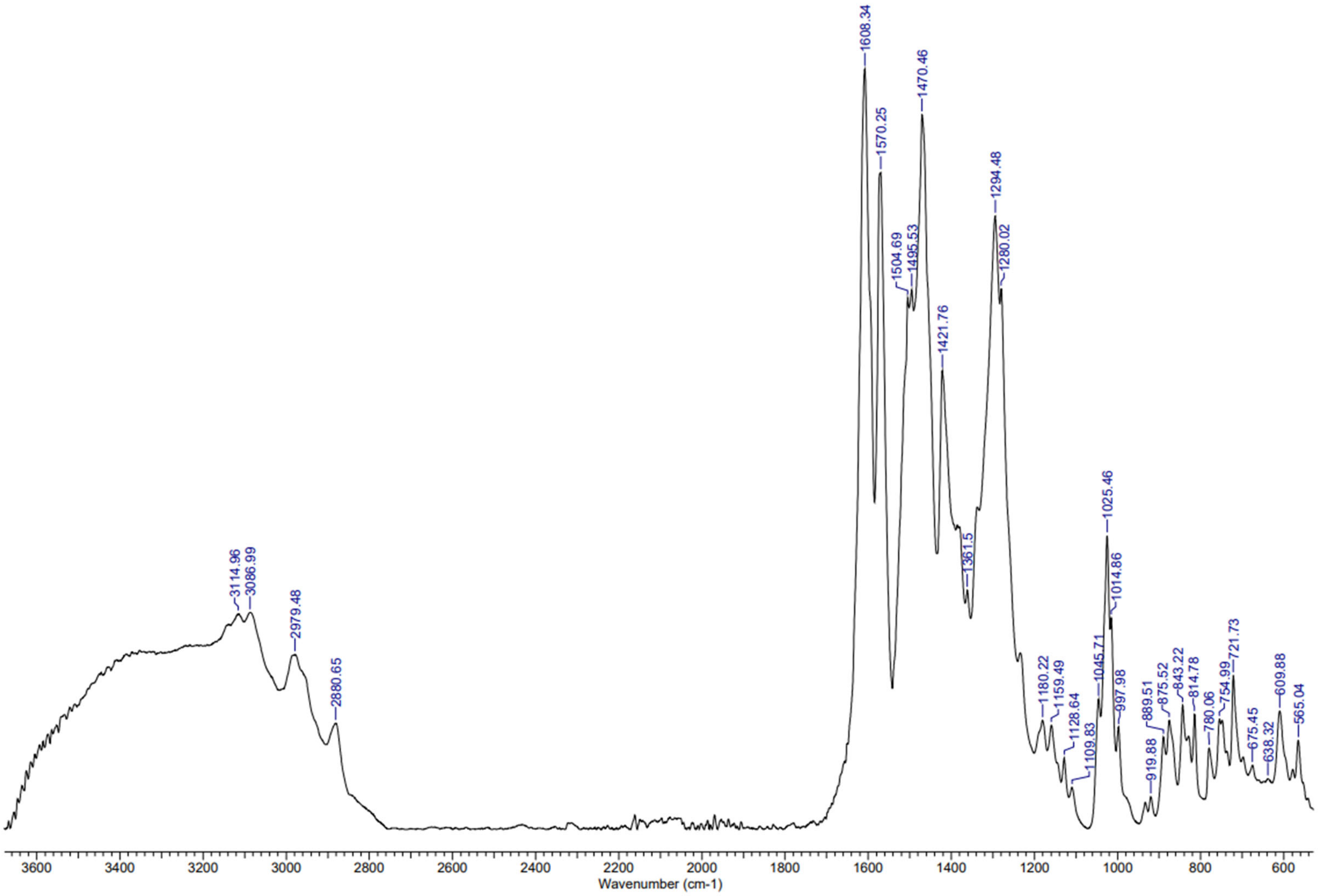

Figure S6. Solid-state IR spectrum of **L3*Lu(NO3)3** at 25ºC

X-ray analysis data

Table S1. Crystallographic data for the ligand **L3** and the complexes **L•Lu(NO$_3$)$_3$** and (**L3**)$_3$Lu$_3$O$_2$(NO$_3$)$_5$.

	**L3**	**L1•Lu(NO3)3**	**L2•Lu(NO3)3**	**L3•Lu(NO3)3**	**(L3)**$_3$Lu$_3$O$_2$(NO$_3$)$_5$
Empirical formula	$C_{22}H_{20}F_2N_4O_2$	$C_{22}H_{24}LuN_7O_{12}$	$C_{24}H_{29}Cl_2LuN_8O_{14}$	$C_{24}H_{23}F_2LuN_8O_{11}$	$C_{66}H_{66}F_6Lu_3N_{17}O_{23}$
Formula weight	410.42	753.45	899.42	812.47	2104.26
Temperature, K	295	295	295	295	100
Crystal system	Orthorhombic	Monoclinic	Triclinic	Monoclinic	Orthorhombic
Space group	P $2_12_12_1$	P 2_1/n	P -1	P 2_1/c	P bcn
Z(Z′)	4 (1)	4 (1)	2 (1)	4 (1)	4 (0.5)
a, Å	8.3819(3)	8.32770(10)	9.6408(4)	8.9589(4)	21.3714(4)
b, Å	10.6432(3)	21.2957(5)	9.7352(4)	16.5501(7)	18.6993(4)
c, Å	22.0479(6)	15.1884(4)	18.0401(6)	19.1104(9)	19.5530(4)
α, °	90	90	84.951(3)	90	90
β, °	90	91.412(2)	79.175(3)	90.760(4)	90
γ, °	90	90	73.680(3)	90	90
V, Å^3	1966.90(10)	2692.76(10)	1594.90(11)	2833.3(2)	7814.0(3)
D_{calc},g cm^{-1}	1.386	1.859	1.873	1.905	1.789
μ, cm^{-1}	8.72	77.03	33.43	74.68	38.59
F(000)	856	1488	892	1600	4128
2θmax, °	134	134	56	134	54
Reflections measured	13287	18334	43553	20022	85879
Independent reflections (R_{int})	3377 (0.0517)	4708 (0.0499)	7533 (0.0616)	4989 (0.1151)	8510 (0.0426)
Observed reflections [$I > 2\sigma(I)$]	2068	4007	5297	2557	7308
Parameters	279	387	470	417	591
R1 with I>2σ(I)	0.0333	0.0356	0.0356	0.0435	0.0318
wR2 (all data)	0.0751	0.0984	0.0522	0.0993	0.0922
GOF	0.855	1.060	0.836	0.808	1.091
$\Delta\rho_{max}/\Delta\rho_{min}$, e Å^{-3}	0.162/-0.116	0.751/-1.739	0.966/-0.912	0.956/-1.336	2.299/-1.144
CCDC	2221666	2221667	2191901	2221670	2232270

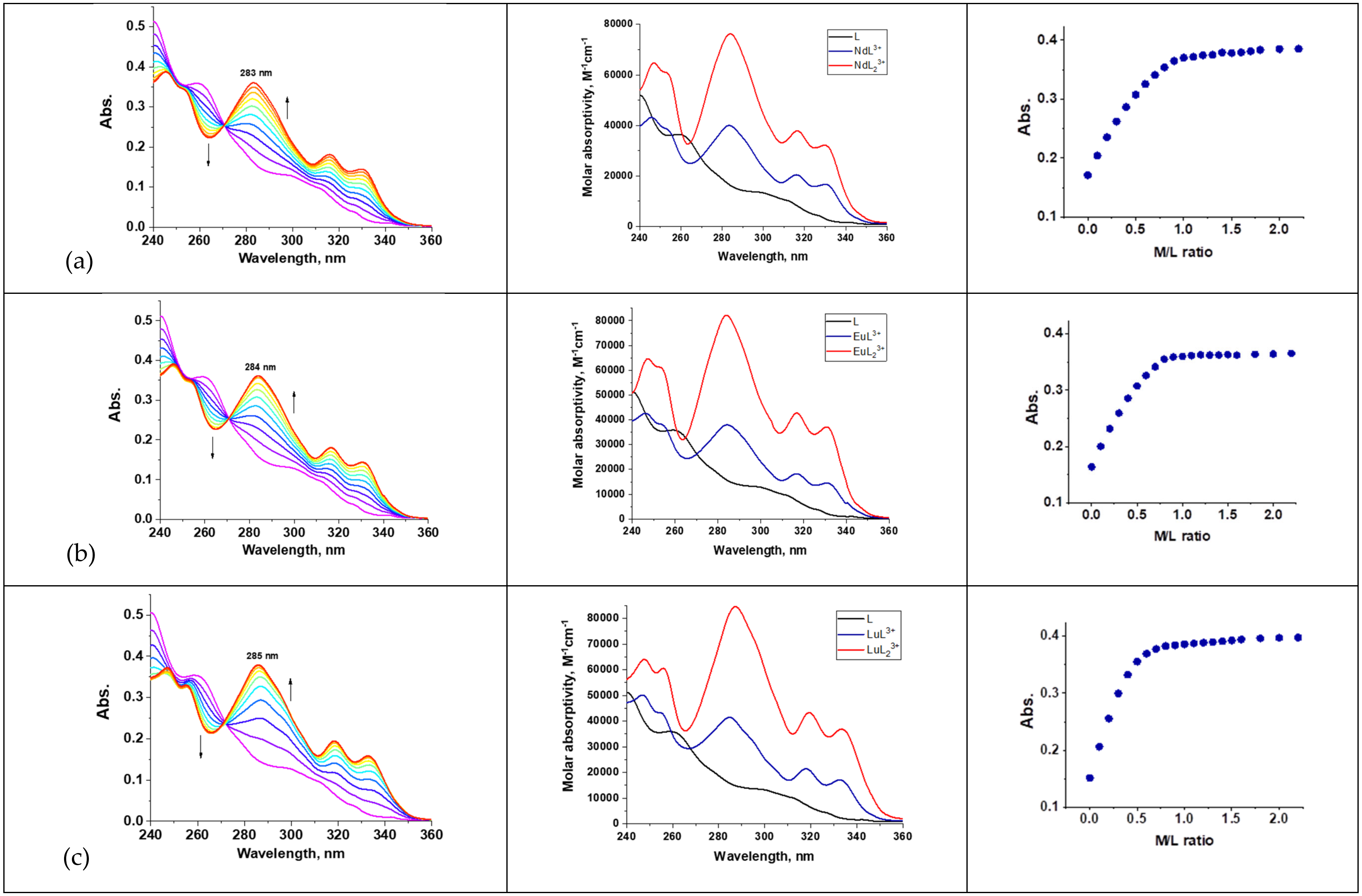

Figure S7. Spectrophotometric titration of **L3** (ca. 10^{-5} mol L^{-1}) with (a) Nd^{3+}, (b) Eu^{3+} and (c) Lu^{3+} ions (ca. 10^{-3} mol L^{-1}) in CH_3CN solution (T = 25.0 ± 0.1 °C, I = 0 M, V_0 = 2.0 mL). Left: absorption spectra; middle: the molar absorptivities of free ligand **L3** and Ln(III) complexes calculated from spectral deconvolution; right: titration curve at maximum absorption wavelength
